# Malignant Fungating Wounds in Locally Aggressive Cutaneous Squamous Cell Carcinoma: A Case Series

**DOI:** 10.7759/cureus.81114

**Published:** 2025-03-24

**Authors:** Tarek Zieneldien, Holly Lam, Sophia Ma, Janice Kim, Ammar Mattar, John Greene

**Affiliations:** 1 Medicine, Johns Hopkins University School of Medicine, Baltimore, USA; 2 Medicine, Morsani College of Medicine, University of South Florida, Tampa, USA; 3 Medicine, College of Osteopathic Medicine, Michigan State University, East Lansing, USA; 4 Vascular Surgery, Edward Via College of Osteopathic Medicine, Auburn, USA; 5 Internal Medicine, Moffitt Cancer Center, Tampa, USA

**Keywords:** immunosuppression, malignant fungating wounds, palliative care, squamous cell carcinoma, supportive care

## Abstract

Despite surgical removal being considered the first-line treatment, managing high-risk squamous cell carcinoma (SCC) cases remains challenging and lacks uniformity as associated complications of less common secondary bacterial infections are underreported. The skin, as well as its appendages, constitute the first line of defense against infectious pathogens. In patients with locally aggressive skin cancers, susceptibility to infectious microorganisms is elevated due to skin lesions and immunosuppression resulting from chemotherapy, surgery, immunotherapy, and stem cell transplantation. Furthermore, immunosuppressed patient populations, when infected, often have extended disease duration and heightened disease burden. Malignant fungating wounds (MFWs), cancerous lesions that typically occur during the terminal stages of a patient’s illness when tumors infiltrate the skin and surrounding tissue, may further complicate cases as they often can be misdiagnosed as abscesses or infections. Due to this, we sought to thoroughly characterize the disease progression, treatment efficacy, and risk factors of two patients with locally aggressive SCCs afflicted with MFWs and secondary *Pseudomonas aeruginosa *(*P. aeruginosa*)infections. We report two cases of cutaneous SCC (cSCC) in immunocompromised patients, one with HIV and the other a lung transplant recipient on chronic immunosuppression. Both cases were complicated by extensive tumor invasion, chronic infection, and multidrug-resistant *P. aeruginosa*. These cases highlight the aggressive nature of cSCC in immunocompromised individuals and the complexities of balancing oncologic treatment, palliative care, and infectious disease management.

## Introduction

Cutaneous squamous cell carcinoma (cSCC) is a common keratinocyte-derived malignancy that arises from the epidermal layer of the skin. It is the second most prevalent type of skin cancer in the United States, following basal cell carcinoma [1]. cSCC is most frequently diagnosed in white patients aged 70 years or older, with its prevalence increasing significantly with age [2,3]. While cSCC generally occurs at a higher rate in men than women, studies indicate that men more commonly develop cSCC on the ears, scalp, and neck whereas women are more likely to develop it on the lower limbs [2,3]. Key risk factors include ultraviolet (UV) light exposure, genetic conditions, and lighter skin tones [3,4]. Immunosuppression is another well-established factor as patients with hematological malignancies, those taking immunosuppressants, and transplant recipients have a higher risk of developing cSCC and face a greater likelihood of metastasis [4,5]. Environmental factors such as arsenic, polycyclic aromatic hydrocarbons, or nitrosamines have also been implicated in the induction of this malignancy [4].

The precursor lesion of cSCC, actinic keratosis, arises from prolonged exposure to UV solar radiation [1]. Although the precise mechanism by which actinic keratosis evolves into cSCC has yet to be fully elucidated, evidence suggests it may be linked to skin dysbiosis, particularly involving an overabundance of *Staphylococcus aureus *(*S. aureus*) [6]. Bacterium-derived toxins may damage DNA, leading to the release of proinflammatory cytokines potentially contributing to carcinogenesis [6]. For instance, a study showed that *S. aureus* secretions applied to human keratinocytes led to increased levels of IL-6, IL-8, and TNF-alpha, key mediators of inflammation [7]. Diagnosis of cSCC typically involves a thorough skin examination followed by a biopsy for histopathological confirmation [8]. In most cases, cSCC treatment is achieved through surgical excision, with Mohs micrographic surgery being the preferred technique due to its high precision and tissue-sparing benefits [9]. Radiation therapy is another option, often used for older patients, those who are poor surgical candidates, or when clear surgical margins cannot be achieved [9].

cSCC can manifest in various ways, including as a rough, red scaly area; an open sore with raised borders; or a wart-like growth. When cSCC presents as an open sore, chronic infection can lead to complications such as osteomyelitis, a serious condition that occurs when the infection spreads to the bone. In severe cases, skull base osteomyelitis (SBO), a fatal condition, can develop when infection spreads from lesions in areas like the sinuses or ears [10]. In one study of 100 patients with skin cancer, the majority of whom had SCC, it was found that the most commonly cultured bacteria from their lesions were *S. aureus* and *Pseudomonas aeruginosa* (*P. aeruginosa*) [11]. Gram-positive bacteria such as *S. aureus* are responsible for over half of the microbiologically documented infections among cancer patients [12]. Cancer therapy and cancer induced-related immunosuppression synergistically increase this patient population’s vulnerability to gram-positive infections, especially methicillin-resistant *S. aureus *(MRSA) [12]. 

*P. aeruginosa*, an opportunistic gram-negative bacterium that causes infections in cancer patients, has been strongly associated with increased tumor sizes in cSCC [13,14]. Similarly to *S. aureus*, surgery, immunotherapy, radiotherapy, stem cell transplantation, and chemotherapy make oncological patients susceptible to infection to these bacteria. Hospital patients are especially vulnerable to acquiring *P. aeruginosa *from reservoirs such as potable water, toothbrushes, and sanitizers. Management of osteomyelitis caused by *P. aeruginosa*, including SBO, typically involves a combination of surgical debridement and a tailored antibiotic regimen. Initial parenteral antibiotics for up to 15 days, followed by oral fluoroquinolones for a total treatment duration not exceeding six weeks, has been shown to be effective [15]. Additionally, a case of a *P. aeruginosa* sternal wound muscle flap coverage following cancer resection with the aforementioned treatment combination has been especially effective, as radiated areas (if the patient has undergone radiation therapy) are prone to poor healing and have higher rates of graft failure [16]. However, despite these approaches, Pseudomonas infections are difficult to treat due to extensive resistance mechanisms such as intrinsic antibiotic resistance, where a bacterium’s membrane permeability to antimicrobials is restricted; efflux systems, which allow the bacterium to move harmful compounds outside its cell membrane; and antibiotic inactivating enzymes [13]. Pseudomonas also has a unique ability to form biofilms that are difficult for the host’s defense system to detect [13]. The presence of virulent bacteria can significantly complicate postoperative outcomes of skin cancer lesions due to the challenging nature of treating these infections as a result of these resistance mechanisms [11].

Malignant fungating wounds (MFWs) are underreported complications of advanced cancers, most commonly affecting the breast, followed by the head and neck region, where they typically emerge in the late stages of the disease course [17]. These wounds, affecting approximately 5-14% of patients an advanced malignancy, develop when cancerous cells like those of cSCC penetrate and proliferate within the skin tissue [18-20]. Malignant tumors of the head and neck frequently involve the skin and underlying tissues, presenting with characteristic features such as a fetid odor, sloughing, and bleeding. The malodor and exudate commonly associated with these lesions are linked to the presence of at least one obligate anaerobic bacterium with a concentration exceeding 105/g bacteria [19]. Microbial metabolites like putrescine and dimethyl trisulfide (DMTS) are associated with the manifestation of these symptoms [21]. As fungating wounds typically signal a terminal prognosis, treatment is mainly palliative, focusing on symptom management rather than curative interventions, as no proven therapies currently exist to fully alleviate symptoms [22]. Tumors breaching the skin and forming a MFW may sometimes be mistaken for an abscess or infection. Due to this, these tumors are often influenced by a multitude of cognitive biases in medical decision making, such as premature closure, Sutton’s slip, and representativeness restraint [23]. Such biases are particularly common in the evaluation of aggressive and locally advanced tumors, further complicating accurate diagnosis and management.

Overall, the rising rates of antimicrobial resistance among both gram-positive and gram-negative bacteria create hurdles for effective treatment [12,13]. Compounding this issue is the limited development of new antibiotics, especially those targeting resistant gram-negative pathogens. MFWs may introduce another layer of complexities in such cases. In this case series, we present two immunocompromised patients with MFWs: the first patient had cSCC on the scalp complicated by osteomyelitis, while the second was lung transplant recipient with a right auricular cSCC, and in both cases, *P. aeruginosa* was cultured from the wounds.

## Case presentation

Case 1

A 52-year-old male with a history of needlestick injury-acquired HIV and cSCCs on his right arm, right leg, and scalp, presented with an infection on his scalp. He started Atripla three years ago and his last recorded CD4 count was 600 with an undetectable viral load. The patient had no opportunistic infections except for thrush several years previously. He did not have a history of sexually transmitted diseases or hepatitis A, B, or C. The patient reported on his February 2014 visit that he was also on prophylactic Azithromycin two times a week for mycobacterium avium-intracellulare, Dapsone 100 mg daily for pneumocystis pneumonia, and Fluconazole 100 mg daily for thrush. He had previously undergone excision biopsy of the scalp lesion twice and 7 weeks of radiation therapy.

**Figure 1:**
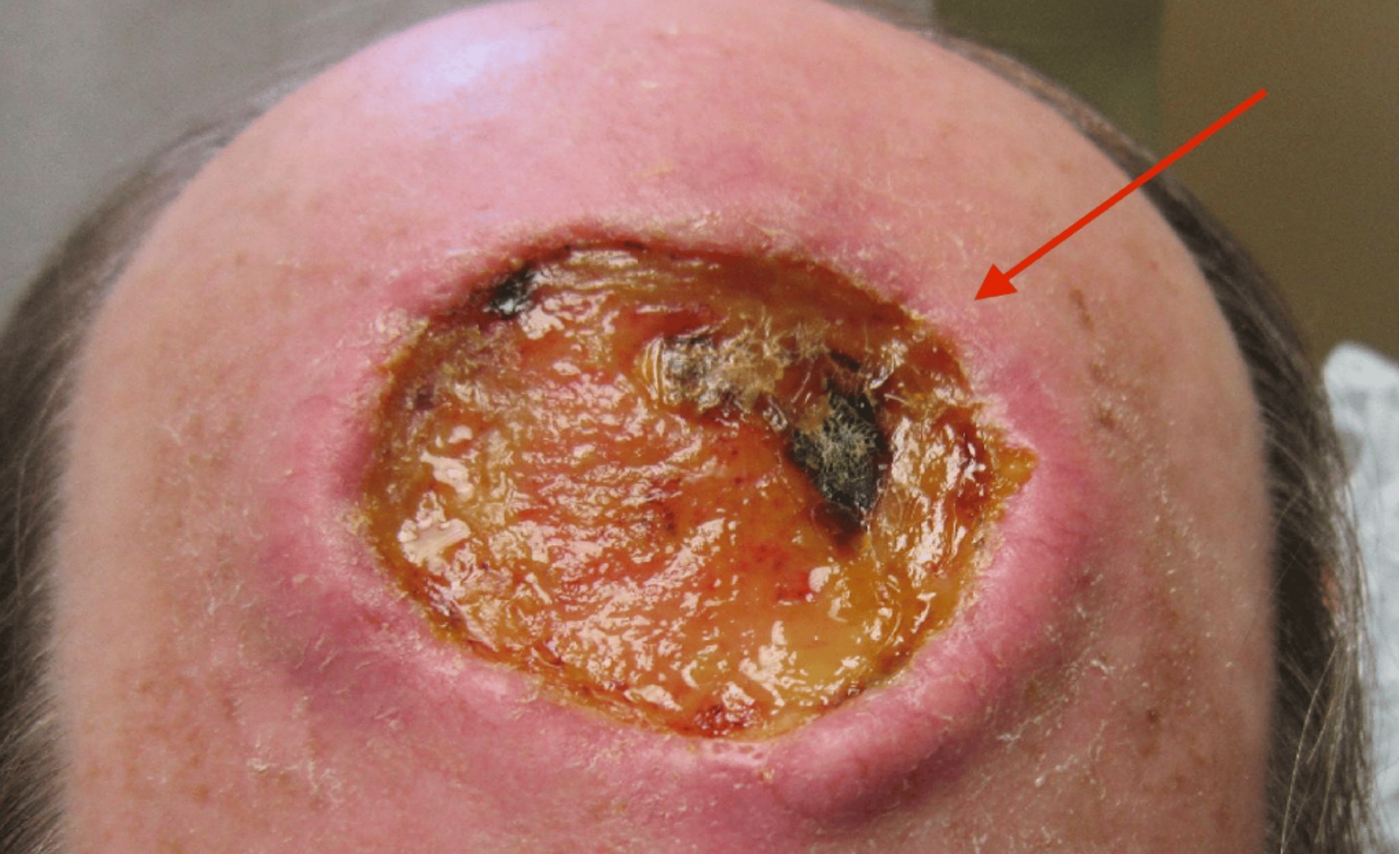
Large SCC of the scalp and osteomyelitis of the skull with a wound infection characterized by an overgrowth of P. aeruginosa and mixed gram-positive flora. The red arrow indicates the ulcerated area with wound infection. SCC: Squamous cell carcinoma

The cSCC on his scalp, which first appeared in May of 2013, had enlarged to a 4-inch-wide ulcer with deep crater. The cSCC had invaded deeper into the bone such that the underlying bone and structures were infected. MRI imaging revealed osteomyelitis of the skull involved frontal calvaria with superior sagittal sinus destruction and soft tissue enhancement, but no thrombosis. Additionally, there was a 7 x 7 cm warm, pink, open wound on the vertex of the scalp with raised firm borders, purulence and a foul odor, which was indicative of an MFW (Figure 1). He was scheduled to have surgical resection with muscle flap closure. A biopsy of the raised border of his scalp lesion confirmed SCC in situ. A culture of purulent discharge from the scalp wound grew 4+ gram-negative flora, 4+ mixed gram-positive flora, and 4+ *P. aeruginosa* bacteria. The *P. aeruginosa* was determined to be susceptible to cefepime (minimum inhibitory concentration (MIC) = 2 µg/mL), ciprofloxacin (MIC ≤ 0.25 µg/mL), gentamicin (MIC = 2 µg/mL), tobramycin (MIC ≤ 1 µg/mL), and piperacillin/tazobactam (MIC = 8 µg/mL) antibiotics. The patient was started on doxycycline antibiotics while scalp discharge culture sensitivity results were pending, and the results later confirmed doxycycline susceptibility.

The patient then underwent a frontal calvarial craniectomy involving oncologic resection with full-thickness bone removal, followed by scalp flap reconstruction as part of a plastic surgery and neurosurgery joint case. Surgery included irrigation and debridement, resection of squamous carcinoma, and a flap and skin graft coverage. The patient was discharged on the evening of postoperative day 1 without incident, prescribed pain medication and levofloxacin at 750 mg for 30 days, and was instructed to keep the surgery dressings dry and his head elevated.

Case 2

**Figure 2:**
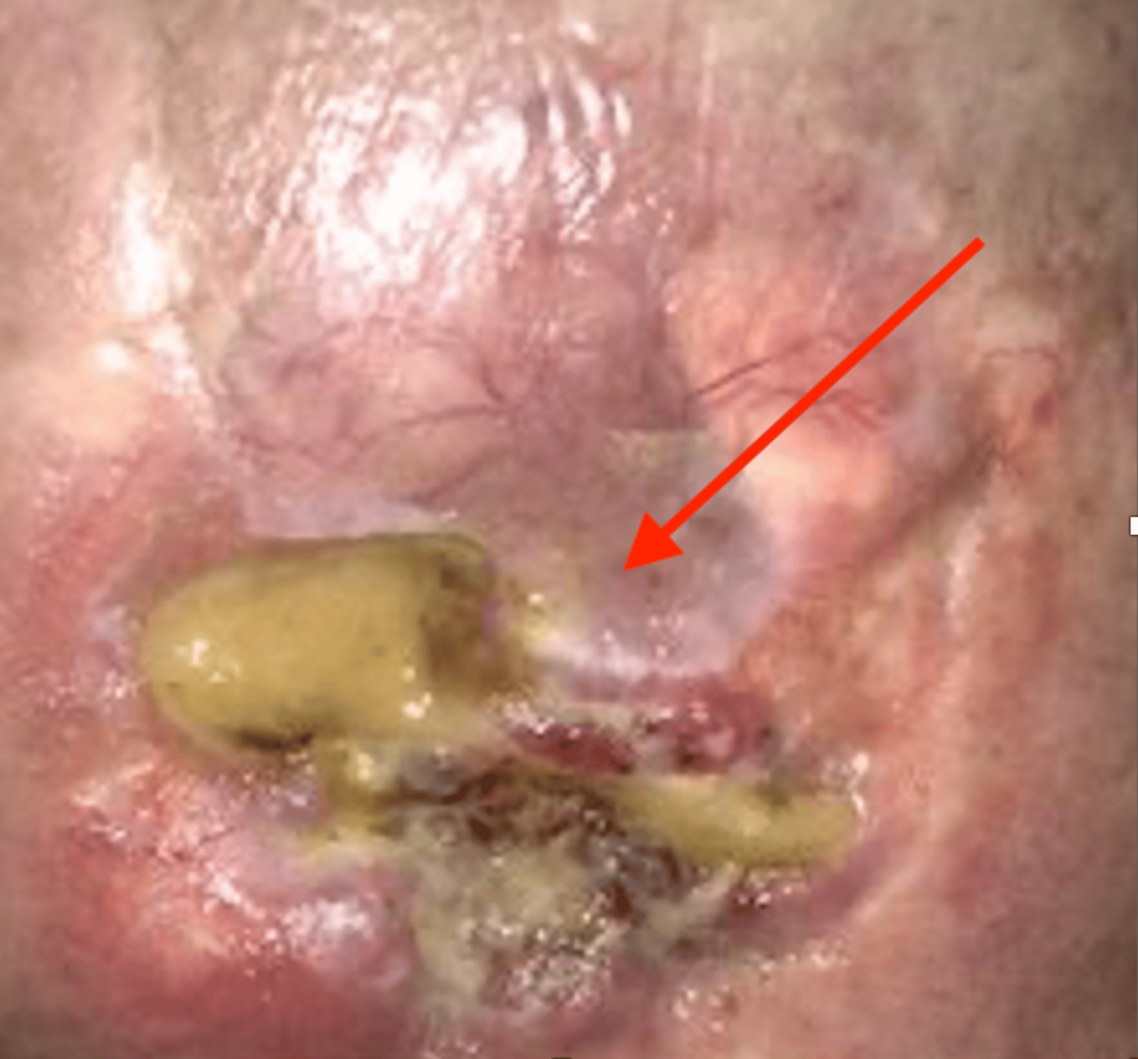
SCC of the right auricular area with a wound infection characterized by P. aeruginosa is indicated by the red arrow. SCC: Squamous cell carcinoma

**Figure 3:**
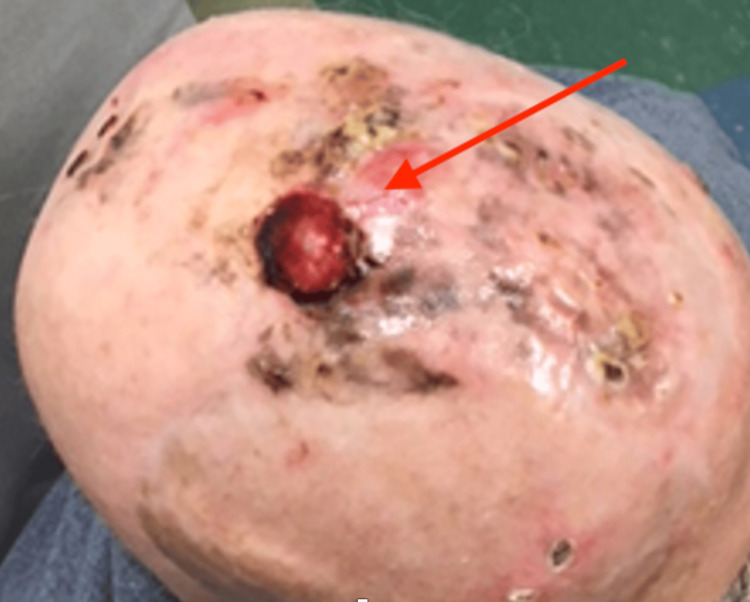
Clear cell SCC of the scalp with ulceration, hemorrhagic blistering, and secondary P. aeruginosa infection is highlighted by the red arrow. SCC: Squamous cell carcinoma

The second cSCC patient was a 67-year-old white man who presented with poor appetite, progressive right facial droop, right ear and head pain, and difficulty chewing. He had a lung transplant in 2011 and had been on chronic immunosuppressive therapy since then. After the lung transplant, the patient subsequently developed multiple cSCCs of the forehead, scalp, and right ear area in October 2016. He had an open wound on the right auricular area consistent with an MFW characterized by yellow exudate, granulation tissue, surrounding redness, and a serous fluid (Figure 2). The top of his scalp had a 1-inch-wide hemorrhagic blister with some areas of excoriation, but no obvious infection. Wound cultures for bacterial, fungal, and acid-fast bacillus (AFB) were obtained from the surface of the right ear area. The wound culture grew 3+ *P. aeruginosa* susceptible to cefepime (MIC = 2 µg/mL), ciprofloxacin (MIC = 0.5 µg/mL), gentamicin (MIC: ≤ 1 µg/mL), tobramycin (MIC: ≤ 1 µg/mL) and piperacillin/tazobactam (MIC = 8 µg/mL). The patient was prescribed 500 mg of ciprofloxacin twice daily for one month to observe any changes to the amount of drainage from the site of infection. This treatment was also given due to a risk of progression to SBO. The patient was started on radiotherapy for his scalp lesion and received 22 Gy until January 2017. Since starting the radiotherapy, the patient developed three new biopsy-confirmed lesions: one on the right lateral cheek showing SCC, one on the mid scalp with clear cell features, and another on the left malar region revealing SCC. The scalp lesion had a poorly differentiated malignant neoplasm with sarcomatoid features extending to the deep tissue base, accompanied by a 1-inch-wide hemorrhagic blister with areas of excoriation (Figure 3). Due to these findings, further radiotherapy was withheld, and the patient underwent surgical resection of the cheek and scalp lesions in January 2017. From the resection, pathology revealed positive margins, with SCC identified at the peripheral margin and peripheral edges.

In February 2017, a new mass lesion was noted over the posterior scalp and was biopsied, revealing poorly differentiated SCC. The patient resumed radiotherapy targeting the ear and scalp lesions, receiving a total dose of 60 Gy with concurrent chemotherapy carboplatin and paclitaxel (six cycles carboplatin and two cycles paclitaxel), completing treatment in August 2017. Subsequently in the same month, the patient underwent palliative tumor debulking of the right external ear, including a complete auriculectomy involving the pinna and conchal bowl. In September 2017, cetuximab therapy was initiated and continued through December 2017, with an initially favorable response. However, recurrent and progressive disease was noted approximately three months after completing therapy. In March 2018, cetuximab was restarted. However, in August 2018 the patient was hospitalized from August 15 to August 22 for altered mental status, urinary tract infection, and acute respiratory failure. Cetuximab was resumed on August 31, 2018, with the final dose administered on October 5, 2018.

In October 2018, the patient was hospitalized again due to failure to thrive and severe pain. During this admission, progression of his locally advanced SCC was confirmed. Given the high risks associated with further invasive procedures, the patient was deemed too high risk for additional surgical procedure due to trismus, morbid factors, and a previous failed intubation attempt. As additional chemotherapy was likely to negative impact his quality of life given his immunocompromised performance status, the patient was transitioned to hospice care for palliative symptom and pain management with weekly therapy.

## Discussion

Even though most cases of cSCCs are effectively treated through surgical excision, advanced cSCC presents significant challenges, including diminished quality of life and increased mortality risk. Prompt and effective management of advanced cSCC is crucial as delayed diagnosis, local invasion, and metastasis can result in higher morbidity, prolonged treatment times, and increased healthcare costs [24]. For localized cSCC, the standard therapy is surgical excision via Mohs micrographic surgery, curettage and electrodessication, or standard excision [25]. Despite surgery being the first-line and most efficacious treatment for cSCC, radiation therapy can be employed in situations where surgery is not an option, contraindicated, or declined by the patient [25].

Our two cases illustrate different clinical trajectories of advanced cSCC complicated by secondary infections, underlying immunosuppression, and MFWs. Case 1 involved a 52-year-old patient with HIV-associated immunosuppression that presented with an extensive SCC on the scalp that had invaded the calvarial bone and caused osteomyelitis. Despite having an undetectable viral load, the lesion progressed locally to full-thickness bone involvement, necessitating a frontal calvarial craniectomy with flap reconstruction. This case elucidated that even in situations where the HIV is well-controlled, advanced cSCC with MFW can cause rapid structural destruction. On the other hand, Case 2 involved a 67-year-old lung transplant recipient on chronic immunosuppression. Over several years, he developed various recurrent cSCCs of the scalp and face. He underwent various treatments including radiation therapy, cetuximab, and chemotherapy, but still experienced progressive SCC with complications including persistent pain, hemorrhagic blistering, and multiple open wounds. His wound care history revealed a chronic reliance on topical agents such as Polysporin and Silvadene, the latter of which he avoided due to the unpleasant odor.

The presence of MFWs in both cases complicated management. In advanced cases of cSCC, particularly when MFWs are present, secondary infections are a concern. These wounds often become colonized by bacteria, contributing to a variety of complications. Among these pathogens, *P. aeruginosa* is notable for its ability to adapt to difficult conditions, allowing it to be broadly distributed in a wide range of habitats [26]. The pathogenicity of this microorganism arises due to resistance to various antibiotic classes and high genome plasticity [27]. The colonization by *P. aeruginosa* in both cases is noteworthy, as it can contribute to delayed wound healing and increased antibiotic resistance. Thus, it is imperative for healthcare providers to remain vigilant and keep a high index of suspicion when dealing with patients who have secondary infections to locally aggressive skin cancer [12]. 

Treatment approaches differed between the cases, reflecting the heterogeneity of advanced cSCC management. While Case 1 was managed primarily with surgery, Case 2 required a multimodal approach. This aligns with current guidelines, which recommend tailoring treatment to individual patient factors and disease characteristics. Treatment may include cancer excision and a combination of surgical debridement and targeted antibiotic therapy [28]. A skin or muscle flap may also be used to close the open defect in cases where there are concerns about cancer recurrence, poor healing, or graft failure, which are often associated with areas that have previously undergone radiation therapy [28].

The ultimate transition to palliative care in Case 2 highlights the potential for poor outcomes in advanced cSCC, particularly in transplant recipients. As MFWs typically occur during the last six months of a patient’s life, they require a primarily palliative approach aimed at managing symptoms, including using antiseptic dressings such as iodopovidone impregnated or silver dressing to minimize fetid odor and infections [29]. Absorbent dressings should also be applied when exudate is present. In the absence of an infection or systemic signs, MFWs should be treated with topical treatment rather than intravenous or prophylactic oral antibiotics [23]. Importantly, if a wound infection is suspected, a swab culture and antibiotic susceptibility testing should be conducted to guide appropriate treatment.

## Conclusions

The cases presented in this study highlight the complex nature of advanced cSCC in immunocompromised patients. Both cases demonstrated the aggressive progression of cSCC, leading to MFWs and secondary infections, particularly with *P. aeruginosa*. The HIV-associated case resulted in extensive local invasion requiring complex surgical intervention, while the transplant-associated case necessitated multiple treatment modalities and ultimately led to palliative care. These outcomes underscore the challenges in managing advanced cSCC in immunosuppressed individuals and the importance of early detection and aggressive treatment. The presence of *P. aeruginosa* in both cases emphasizes the need for targeted antibiotic therapy based on culture and sensitivity results. To improve patient outcomes, healthcare providers can implement preventative measures and adopt evidence-based treatment such as including a combination of superficial debridement, targeted antibiotic monotherapy, and muscle flap coverage for secondary complications associated with cSCC, including MFWs, osteomyelitis, and *P. aeruginosa* infections. The management of these complex cases requires a multidisciplinary approach, incorporating surgical intervention, radiation therapy, chemotherapy, infection control, and wound management as appropriate. Ultimately, these cases demonstrate that advanced cSCC in immunocompromised patients often leads to significant morbidity and may require a transition to palliative care to manage symptoms and improve quality of life.
